# Self-management assessment tools for people with hypertension: a scoping review

**DOI:** 10.1186/s12882-025-04134-y

**Published:** 2025-04-30

**Authors:** Li Mengjiao, Zhao Xujie, Jiang Ping, Huang Liwen, Ning Yuping, Zhang Yangjing, Yan Jianjun

**Affiliations:** 1 Shanghai University of Traditional Chinese Medicine, Shanghai, 201203 China; 2 Shihezi University, Xinjiang Uygur Autonomous Region, Shihezi City, 832000 China; 3 Shanghai Pudong New District People’s Hospital, Shanghai, 200120 China; 4 HuaiHe Hospital of Henan University, Kaifeng, Henan Province 475000 China

**Keywords:** Hypertension, Self-management, Assessment tools, Scoping review

## Abstract

**Background:**

This is a scoping review of the evidence for the use of the Patient Self-Management Assessment Tool for Hypertension (PAT) in people with hypertension. This review examines the content features, reliability, and validity of the PAT for people with Hypertension, as well as contextual and environmental evidence for the tool implementation in clinical practice.

**Objective:**

To synthesize and evaluate the self-management assessment tools available for people with hypertensive, and to guide healthcare professionals in selecting appropriate tools.

**Methods:**

A systematic search was conducted across nine databases, including PubMed, Web of Science, Scopus, Embase, Cochrane Library, CNKI (China National Knowledge Infrastructure), VIP (VIP Information Database), CMB (China Biology Medicine disc) and Wanfang, from their inception to August 17, 2024. The authors extracted and analyzed self-management assessment tools developed for people with hypertension, using a scoping review approach to report the findings.

**Results:**

A total of 41 papers were identified, which reported on 20 assessment tools. These included 12 multidimensional assessment tools for assessing self-management and 8 unidimensional assessment tools for measuring adherence to self-management practices.

**Conclusion:**

The quality of self-management assessment tools for people with hypertension varies widely. There is a need to develop tailored tools for different patient populations to accurately assess self-management capabilities, design intervention strategies, and enhance patient engagement in hypertension management.

**Supplementary Information:**

The online version contains supplementary material available at 10.1186/s12882-025-04134-y.

## Introduction

Hypertension, a prevalent chronic non-communicable disease, is a significant risk factor for cardiovascular diseases and is currently the leading cause of mortality in China [1]. Approximately 970 million individuals worldwide have hypertension [2], a chronic illness that affects between 25 and 35% of adults in 2012–2015 [3]. An enormous burden is placed on families and society at large due to the startling 10.8 million annual deaths associated with hypertension-related diseases [4]. With rapid globalization leading to significant demographic changes, hypertension has become a key risk factor for the burden of disease in people over 50 years old worldwide [5]. Additionally, it is now the second most significant non-communicable risk factor for people between the aged 25–49, contributing to ischemic heart disease, stroke, other cardiovascular diseases, chronic kidney disease, and dementia [6]. Hypertension exerts a multifactorial and complex financial impact on both individuals and society as a whole, highlighting its status as a significant worldwide public health concern [1, 6, 7].

In China, the situation is particularly concerning, with hypertension affecting an estimated 250 million people, and over half of the adult population exhibiting blood pressure levels exceeding the normal range [7]. This makes Hypertension one of the most pressing health challenges facing the country.

Investigative findings indicate that the blood pressure control rate among people with hypertension in China stands at 67.72% [8]. Self-management refers to an individual’s ability to manage their own health status. In the context of chronic diseases, self-management involves proactive and collaborative actions undertaken by chronic disease individual with their social networks and healthcare providers to achieve treatment adherence and prevent disease progression [9, 10]. Previous research has identified various factors influencing self-management behaviors among people with hypertension, which can be categorized as follows: sociodemographic factors, lifestyle factors, disease-related factors, psychosocial factors, and other factors. (1) Sociodemographic factors encompass age, gender, place of residence, educational level, economic status, and Body Mass Index (BMI) [11–15]. (2) Lifestyle factors include smoking, alcohol consumption, and the frequency of blood pressure monitoring [16–18]. (3) Disease-related factors consist of the duration of hypertension, comorbid conditions, the severity of hypertension, and family history [19, 20]. (4) Psychosocial factors involve disease perception, personality traits, and health literacy [20, 21]. Chinese literature on hypertension self-management emphasizes the importance of self-management for hypertensive patients: The ‘*China Long-Term Plan for the Prevention and Control of Chronic Diseases (2017–2025)*’ [22] advocates for the public to consciously adopt a healthy lifestyle and engage in reasonable self-management; while the ‘*Chinese Guidelines for the Prevention and Treatment of Hypertension (Revised 2018)*’ [23] states that all hypertensive patients should participate in self-management to varying degrees. The release of these two documents underscores the necessity of strengthening self-management among hypertensive patients and confirms the significant role of self-management in the prevention and treatment of hypertension [24]. At present, there is no review that comprehensively systematically reviews or compares these self-management scales in terms of reliability, validity, theoretical basis, and other aspects. Therefore, it seems essential to identify and assess the characteristics of self-management among hypertensive patients before implementing intervention studies. However, the self-management level of Chinese hypertensive patients remains sub-optimal, with prevalence, awareness, treatment, and control rates of 61.1%, 51.6%, 45.8%, and 16.8% respectively, which were significantly lower than those in developed Western countries [25, 26].

Comprehensive and accurate assessment of patients’ self-management levels is fundamental for hypertension management [27]. Numerous self-management tools are available to assess hypertensive patients’ self-management level. However, most self-management tools lack a theoretical framework, psychometric quality, and reliability and validity, and focusing narrowly on medication adherence or hypertension-related knowledge. Therefore, it is crucial to choose an assessment tool that can evaluate a person’s self-management from multiple dimensions. The present study employed the scoping review reporting framework developed by Arksey [28] to comprehensively collect hypertensive patients’ self-management assessment tools, aiming to provide healthcare professionals with more comprehensive, scientific, and effective tools.

## Materials and methods

### Defining research questions

We conducted a scoping review based on the Joanna Briggs Institute (JBI) framework [29]. This study is reported according to the PRISMA-ScR (Preferred Reporting Items for Systematic Reviews and Meta-Analyses extension for Scoping Reviews) extension of the scoping review [30]. Our research questions were:

① What self-management assessment tools for hypertension have been developed domestically and internationally? ② What are the reliability, validity, and characteristics of these tools? What is the current status of application and development for self-management assessment tools for hypertension in China?

### Database search

Nine databases (Chinese and English) were searched: CNKI (China National Knowledge Infrastructure), VIP (VIP Information Database), Wanfang, CMB (China Biology Medicine disc), PubMed, Web of Science, Scopus, Embase and Cochrane Library. The search covered the period from their inception until August 17, 2024.

### Search strategy

**Fig. 1:**
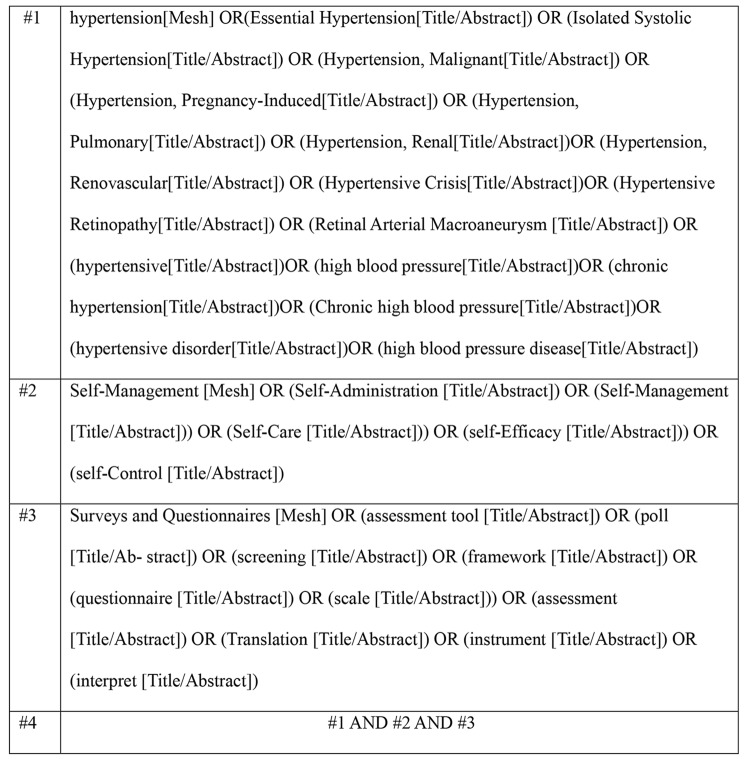
PubMed search strategy

The search was conducted using a combination of subject headings and free-text terms. In both Chinese databases and English databases, the subject headings included “hypertension,” “hypertensive,” “hypertensive diseases,” “chronic hypertension,” “high blood pressure,” “self-management,” “self-care,” “self-control,” “self-management,” “questionnaire,” “scale”, “tool,” “instrument,” “Sinicization,” and “translation.” The search strategy for PubMed is illustrated in Fig. 1.

### Inclusion and exclusion criteria

The inclusion criteria in this study were: ① Study participants aged ≥ 18 years; ② Assessment tools designed for people with hypertension; ③ Original studies reporting on the development, validation, revision, localization, or application of hypertension self-management assessment tools. However, non-English and Chinese peer-reviewed publications, conference abstracts and unavailable full texts were excluded from this review.

### Literature screening and data extraction

Two researchers (ZXJ and LMJ) independently imported the retrieved literature into EndNote 20.6, deduplicated, and screened against the inclusion and exclusion criteria. Disagreements during screening were resolved through consultation with a third researcher (JP) to reach consensus on inclusion. Three researchers (HLW, NYP, and ZYJ) independently analyzed the final included literature, extracting information such as country, time, target population, tool name, scale dimensions, and scale items. Risk of bias was not assessed [29].

## Results

### Literature screening results

**Fig. 2:**
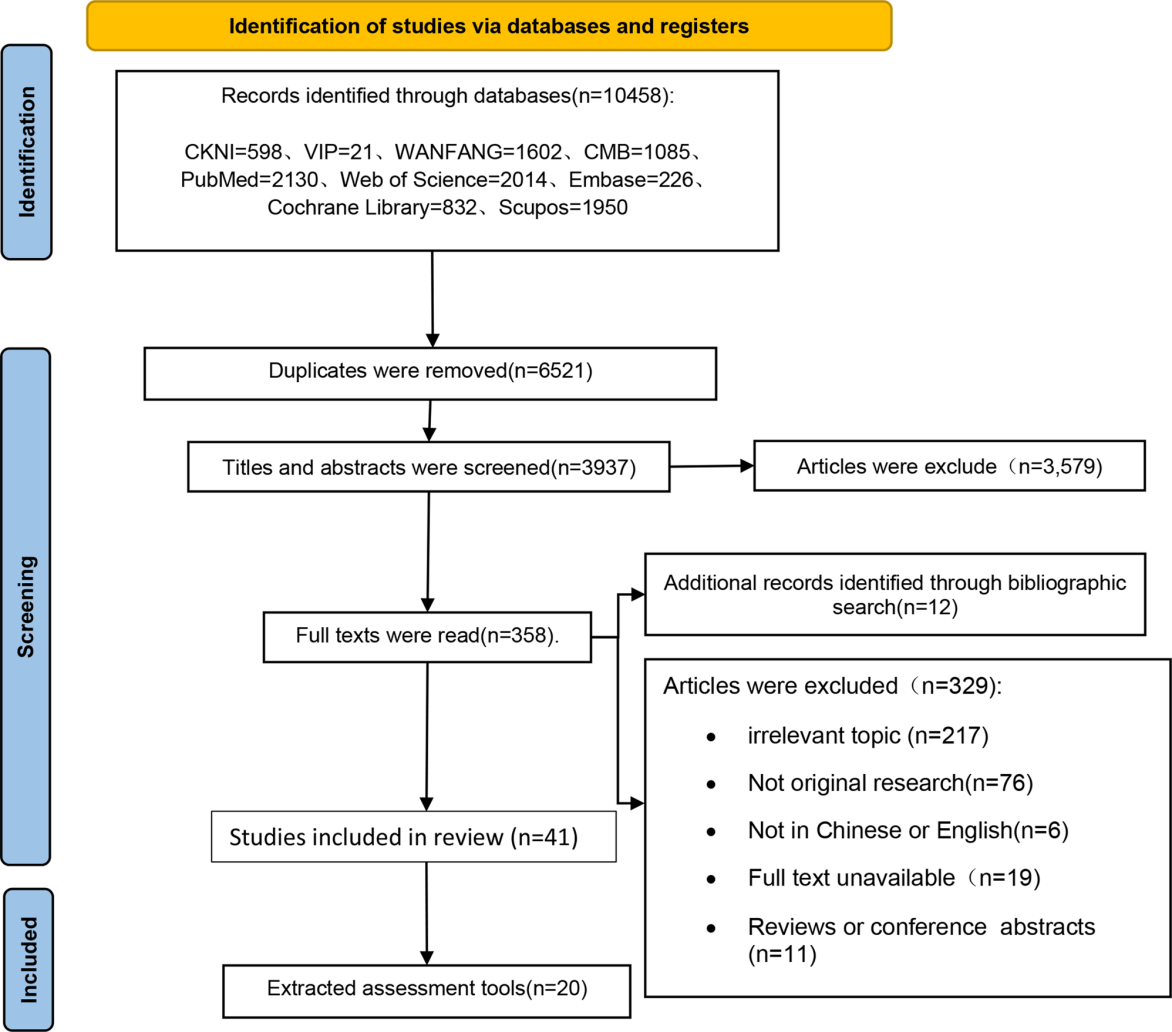
Literature screening process

The initial search yielded 10,458 articles, and an additional 12 articles were identified through reference list tracking. After strict screening based on the inclusion and exclusion criteria, 41 articles were finally included in the assessment tool screening process. Among these 41 articles, 20 assessment tools [31–50] related to the development and validation of self-management assessment tool for hypertension were identified and included in this review. Of these 20 tools, 18 were related to the translation and adaptation of assessment tools. The literature screening process and results are presented in Fig. 2.

### Basic characteristics of self-management assessment tools for hypertensive patients (Table 1)

Among the included tools, four [31, 43, 44, 49] were developed between 1979 and 2000, two [35, 48] between 2001 and 2010, ten [32–34, 38, 40–42, 46, 47, 50] between 2011 and 2020, and four [36, 37, 39, 45] between 2021 and 2023. The target populations varied, with four tools for elderly patients, two for young and middle-aged adults, and two for female patients. The theoretical foundations differed, with five tools based on Orem’s Self-Care Theory, three based on adherence definitions, and three on self-management definitions. Sample sizes varied considerably; ten studies had more than 200 participants, eight studies had 100–200 participants, one study had fewer than 100 participants, and one study did not report the sample size. Four tools did not use the Likert scoring method, while 10 tools used a 5-point Likert scale.

**Table 1:** Characteristics of included studies

Tool name	Country	Year developed	Target population	Construction method	Theoretical basis	Sample size	Evaluation method
Exercise of self-care agency [31]	US	1979	Adults	①②③	Orem’s self-care deficit theory	153	Likert5
The hypertension self-care activity level effects (H-SCALE) [32]	Iran	2019	Hypertensive patients	①②③	Definition of hypertension self-management	293	Likert5
Self-care of hypertension inventory SC–HI [33]	US	2017	Hypertensive patients	①②③	Middle-range theory of chronic illness self-care	193	Scored 1–4
HBP-SCP [34]	US	2014	Elderly hypertensive patients aged 60 and above	①②③	Orem’s self-care deficit theory	213	Likert 4
Maastricht Utrecht adherence in hypertension (MUAH)-questionnaire [35]	Netherlands	2006	Hypertensive patients with at least 1 year of disease history	①②③	Definition of adherence	255	Likert 7
Self-management capability, support, motivation-behaviour scale for elderly hypertension [36]	China	2023	Elderly hypertensive patients	①②	COM-B theoretical model	430	Likert 5
The Hippocratic hypertension self-care scale [37]	Greece	2023	Patients with arterial hypertension	①②③	Definition of hypertension self-management	202	Likert 4
Therapeutic adherence scale for hypertensive patients (TASHP) [38]	China	2011	Patients with primary hypertension who have been taking anti-hypertensive drugs for more than 2 months	①②③	Orem’s self-care deficit theory	594	Likert 5
Questionnaire on self-management behaviors for middle-aged and young hypertensive patients [39]	China	2021	Middle-aged and young hypertensive patients	①②	Definition of hypertension self-management	150	Likert 5
Health self-management ability assessment scale for prehypertensive population [40]	China	2014	Population with elevated blood pressure	①②	Bandura’s social cognitive theory	202	Likert 5
Self-management behavior assessment scale for hypertensive patients [41]	China	2012	Hypertensive patients	①②③	Self-management theory by US scholars Corbin and Strauss [51]	810	Scored 1–3
Hypertensive patient self-management scale [42]	China	2015	Hypertensive patients	①②	Chronic disease self-management theory	151	Likert 5
The appraisal of self-care agency scale-revised ASAS-R [43]	Netherlands	1986	Elderly aged 65 and above	①③	Orem’s self-care deficit theory	140	Likert 5
The self-care ability scale for elderly SASE [44]	Norway	1996	Elderly aged 65 and above	①③	Orem’s self-care deficit theory	57	Likert 5
CoNOCiTHE [45]	Columbia	2023	Hypertensive patients during pregnancy	①②	NOC nursing outcomes classification system		Likert 5
PAG-DT2 + HTA [46]	Mexico	2017	Type II diabetes and hypertensive patients	②③	Concepts of diabetes self-management	145	Items 1–4 scored 1–7; Items 5–8 scored 1–5
The treatment adherence questionnaire for patients with hypertension (TAQPH) [47]	China	2012	Hypertensive patients	①②③	Definition of adherence	278	Likert 4
The ‘‘compliance of hypertensive patients’’ scale (CHPS) [48]	Suomi	2003	Hypertensive patients for a continuous year	①②③	Definition of adherence	150	Scored 1–5
The hill-bone compliance to high blood pressure therapy scale (hill-bone) [49]	US	2000	Hypertensive patients	①②③	Orem’s self-care deficit theory	480	Likert 4
The Facilitators of and barriers to adherence to hypertension treatment scale (FATS) [50]	US	2015	Low-income, black female hypertensive patients	①②③	Orem’s self-care deficit theory	147	Likert 4

*Note* ① Literature retrieval; ② Expert review; ③ Patient or parent interview

### Reliability, validity, and content of hypertension self-management tools

This study’s results demonstrate the excellent reliability of 18 research tools, with the exception of PAG-DT2 + HTA [46] and SASE [44], all of which had Cronbach’s α coefficients of less than 0.7. CHPS [48] used Theta reliability analysis, which differs from other reliability analyses. Six tools underwent intra-class correlation coefficient analysis, but the ICC of the Hippocratic Hypertension Self-Care Scale [37] was 0.653 (<0.75, the standard value). Eleven tools were tested for content validity, with ASAS-R [43] having a significantly higher CVI of 0.976 compared to other assessment tools. Eight assessment tools were analyzed for construct validity, with a tool [37] having a KMO < 0.7, while the Self-Management Behavior Assessment Scale for Hypertensive Patients [41] had a KMO > 0.9, indicating good construct validity. TASHP [45] and the Self-Management Scale for Hypertensive Patients [42] underwent test-retest reliability analysis, which helped establish the specificity and reliability of the tools. These five assessment tools [31, 34, 36, 41, 45] not only have a large number of items, but also the items are generally lengthy, which could be time-consuming and cause discomfort for patients, increasing the likelihood of inaccurate and incomplete data. The most widely used scale in China is the Exercise of Self-Care Agency [31], while SASE [44] is currently the most widely used scale in the Nordic region. Three tools [31, 38, 41] are primarily used for hospitalized hypertensive patients (see Table 2).

**Table 2:** Basic characteristics of hypertension self-management assessment tools

Tool name	Number of dimensions \Items (n)	Dimension Items	Time of assessment	Reliability and validity testing	Characteristics
Exercise of self-care agency [31]	4\43	Proactive and passive response to situations (11 items), motivation (11 items), knowledge base (9 items), and self-concept (12 items)	1 W	Cronbach’s α coefficient = 0.71–0.81; ICC = 0.77–0.81	1. A general tool used to investigate self-care abilities in adults. 2. It contains too many items, requiring a long time to complete. 3. This is the most widely used measurement tool in China. 4. Reliability of 0.91. one week after hospitalization
The hypertension self-care activity level effects (H-SCALE) [32]	5\16	Follow-up (3 items), healthy lifestyle (5 items), increased cognition (4 items), medication therapy (2 items), and other recommendations (2 items)	1 W	Cronbach’s α coefficient = 0.833; Cronbach’s α coefficients for dimensions = 0.60–0.74 ICC = 0.952; CVI = 0.69	1. Primarily used for hypertensive patients. 2. The scale is concentrated in urban areas, with regional limitations. 3. No test-retest validation was performed; subsequent need to expand sample size; and regional test-retest validation
Self-care of hypertension inventory SC–HI [33]	3\23	Self-care maintenance (11 items). Self-care management (6 items) and self-care confidence (6 items)	1 M	CVI = 0.96 Cronbach’s α coefficient = 0.833	1. A specialized tool for hypertensive patients. 2. Small sample size; no validity testing was performed. 3. Widely used, with strong generalizability, and psychometric testing has been conducted
HBP-SCP [34]	3\60	Behavior (20 items), motivation (20 items), and self-efficacy (20 items)	2 W	CVI = 0.92 Cronbach’s α coefficients = 0.83–0.93	1. A specialized tool for elderly hypertensive patients. 2. It was established based on the Hill-Bone Compliance Scale and the Morisky Medication Adherence Scale. 3. It contains many items, requiring a long time to complete
Maastricht Utrecht adherence in hypertension (MUAH)-questionnaire [35]	4\25	Towards healthcare and medication therapy (8 items); lack of self-discipline (6 items); aversion to medication (5 items); active coping with health issues (6 items)	1 M	CVI = 0.86 Cronbach’s α coefficients = 0.63–0.85	1. A specialized tool used primarily to assess medication adherence in hypertensive patients. 2. Other adherence measurement standards used to assess convergent validity may be invalid
Self-management capability, support, motivation-behaviour scale for elderly hypertension [36]	4\33	Capability (10 items), support (7 items), motivation behavior (6 items), and behavior dimension (10 items)	2 W	CVI = 0.94 Cronbach’s α coefficients = 0.701–0.867; KMO value = 0.823; ICC = 0.894	1. A specialized tool mainly used for elderly hypertensive patients (>65 years old). 2. It contains many items and requires a long time to complete
The hippocratic hypertension self-care scale [37]	7\18	Medication (5 items), diet (6 items), exercise (1 item), alcohol (2 items), smoking and blood pressure measurement (1 item each), and appointment adherence (2 items)	–	CVI = 0.807 Cronbach’s α coefficients = 0.591–0.807 KMO value = 0.653; ICC = 0.653	1. This specialized tool is primarily used for patients with arterial hypertension. 2. The tool assesses if patients adhered to self-management practices within the previous month. 3. The electronic scale cannot be used independently
Therapeutic adherence scale for hypertensive patients (TASHP) [38]	4\25	Medication adherence behavior (5 items), adverse medication behavior (8 items), daily life management behavior (10 items), and smoking and alcohol habit management behavior (2 items)	1 M	Cronbach’s α coefficients = 0.827–0.894; KMO value = 0.83; ICC = 0.958	1. A specialized scale mainly used for hospitalized hypertensive patients
Questionnaire on self-management behaviors for middle-aged and young hypertensive patients [39]	4\29	Disease management (8 items), daily life management (8 items), emotion management (6 items), and exercise management (3 items)	–	CVI = 0.653–0.818 Cronbach’s α coefficient = 0.93; KMO value = 0.811	1. A specialized scale mainly used for middle-aged and young hypertensive patients. 2. Small sample size
Health self-management ability assessment scale for prehypertensive population [40]	6\27	Emotional self-management (5 items), exercise self-management (4 items), dietary self-management (5 items), health beliefs (4 items), environmental self-management (5 items), and self-efficacy (4 items)	–	CVI = 0.653–0.87 Cronbach’s α coefficients = 0.792–0.903; KMO value = 0.821	1. A general scale mainly used for populations with elevated blood pressure and at risk of hypertension
Self-management behavior assessment scale for hypertensive patients [41]	6\33	Dietary management (10 items), medication management (4 items), emotion management (7 items), work and rest management (5 items), exercise management (4 items), and condition monitoring (3 items)	–	CVI = 0.82–0.94 Cronbach’s α coefficients = 0.757–0.914; KMO value = 0.904	1. A specialized scale mainly used for hypertensive patients. 2. The scale has regional limitations and contains many items, requiring a long time to complete. 3. No test-retest reliability measurement was performed for the scale
Hypertensive Patient Self-Management Scale [42]	4\21	Treatment management dimension (8 items), diet and exercise management dimension (5 items), lifestyle management dimension (5 items), and risk factor management dimension (3 items)	15d	CVI = 0.875–1; Cronbach’s α coefficient = 0.854; KMO value = 0.703; ICC = 0.767–0.870	1. A specialized scale mainly used for hypertensive patients. 2. It focuses on patients’ management of their condition in daily life
The appraisal of self-care agency scale-revised ASAS-R [43]	3; \15	Self-care ability (6 items), developing self-care ability (5 items), and lack of self-care ability (4 items)	Present	CVI = 0.976 Cronbach’s α coefficients = 0.89–0.90	1. A specialized scale mainly used for elderly hypertensive patients. 2. It emphasizes self-care behaviors, observation, judgment, decision-making, and execution abilities. 3. It has been translated, used, and validated in multiple countries
The self-care ability scale for elderly SASE [44]	3\17	Goals (8 items), environment (2 items), and skills (7 items)	–	Cronbach’s α coefficient = 0.68	1. A specialized scale mainly used for elderly hypertensive patients. 2. No validity validation or test-retest reliability was performed; this is mainly used extensively in Nordic countries. 3. Evaluates patients’ ability to manage blood pressure in daily life
CoNOCiTHE [45]	2\72	Knowledge of disease processes; hypertension risk control; 19 questions; 72 items in total	During pregnancy	Cronbach’s α coefficient = 0.92	1. A specialized tool mainly used for hypertensive patients during pregnancy. 2. Few participants; no sample survey among patients; no reliability and validity testing. 3. It contains many items; no simplification was performed
PAG-DT2 + HTA [46]	4\20	Adherence; coping with illness and difficulties; confidence and self-efficacy; coping or obstacles, 9 questions. Blood glucose monitoring (2 items), blood glucose control (2 items), healthy diet (1 item), physical activity (3 items), coping (5 items), weight maintenance (1 item), confidence in diabetes management (3 items), and items 8 and 9 on coping outcomes (2 items)	1 W	Cronbach’s α coefficient = 0.561	1. A general tool mainly for patients with hypertension and diabetes complications. 2. There are many influencing factors for the scale
The treatment adherence questionnaire for patients with hypertension (TAQPH) [47]	6\28	F1 (“medication”, 9 items), F2 (“diet”, 9 items), F3 (“stimulants”, 3 items), F4 (“weight control”, 2 items), F5 (“exercise”, 2 items), and F6 (“stress relief”, 3 items)	1 M	Cronbach’s α coefficients = 0.72–0.94; KMO value = 0.83	1. A specialized tool mainly for evaluating the treatment effects in hypertensive patients
The ‘‘compliance of hypertensive patients’’ scale (CHPS) [48]	5\13	Lifestyle (3 items), intent (4 items), attitude (3 items), responsibility (1 item), and smoking (1 item)	1 M	Theta coefficient = 0.80	1. A specialized tool mainly used for patients with a 1. year history of hypertension. 2. It only evaluates changes in the patient’s condition over the past week
The hill-bone compliance to high blood pressure therapy scale (hill-bone) [49]	3\14	Medication adherence (3 items), regular follow-up (2 items), and salt intake (9 items)	–	Cronbach’s α coefficients = 0.74–0.84	1. A specialized scale mainly used to assess treatment adherence in patients. 2. High-specificity, simple items
The facilitators of and barriers to adherence to hypertension treatment scale (FATS) [50]	4\18	Social support (4 items), positive behaviors to improve treatment adherence (6 items), factors hindering treatment adherence (5 items), and hypertension knowledge (3 items)	1 M	Cronbach’s α coefficients = 0.64–0.81	1. A specialized scale mainly used for low-income, black female hypertensive patients. 2. Used to assess factors influencing adherence to hypertension treatment. 3. No reliability and validity testing was performed; it was mainly used for measurement in the black population

*Note* Cronbach’s α coefficient for reliability testing; Kaiser Meyer Olkin (KMO) for structural validity; Content Validity Index (CVI) for content validity; Interclass Correlation Coefficient (ICC) for intraclass correlation coefficient

## Discussion

### Hypertension self-management assessment should comprehensively consider complexity and multidimensionality

After extensive investigation and study, it has been discovered that self-management assessment instruments for hypertension are complex and require analysis from various perspectives, requiring analysis and evaluation from a range of angles, including medication use, disease cognition, and emotion [52]. Out of the 20 assessment tools included in this study, the most commonly used ones focused on medication adherence, lifestyle management, disease management, and knowledge of the disease, with medication adherence and lifestyle assessment being the most prominent dimensions. On the other hand, emotion management, exercise management, and the disease-related cognitive status received relatively little attention, and the only assessment tools which included social support and influencing factors were the individual ones. A total of 17 tools specifically designed for the self-management of patients with chronic hypertension were selected in this study. The HBP-SCP is the most widely used hypertension assessment tool. It serves as a specific tool for elderly hypertensive patients. Moreover, it is a unidimensional scale, which is used to assess the self-management habits of hypertensive patients [53]. There are six multidimensional and all-encompassing assessment tools for self-management assessment of chronic hypertensive patients, but three tools [32, 39, 41] have small sample sizes and geographical limitations. Expanding the sample sizes and further validating the utility of the tools are recommended in the future. The TASHP Scale is recommended for assessing self-management in hospitalized hypertensive patients; the Hippocratic Hypertension Self-Care Scale is mainly used by community healthcare workers to assess the self-management of blood pressure in chronic hypertensive patients who have been living at home for a long period of time; the scale compiled by Brokalaki [37], which is suitable for arterial hypertensive populations; the TASHP, the Hippocratic Hypertension Self-Care Scale, and the Hippocratic Hypertension Self-Care Scale, which not only provide a multidimensional and comprehensive assessment of the hypertensive population, but also allow for the use of different assessment tools for different populations, suggesting that the future use of personalized self-management assessment tools for self-management of hypertensive disorders caused by different populations and disease types can enable healthcare professionals to adopt a more effective and personalized plan for the intervention and treatment of hypertension.

### Evaluation tools from China still need to be improved

Among the evaluation tools included in this study, the tools numbered [31–35, 37, 38, 47–50] were all constructed through the methods of literature retrieval, expert review, and patient interviews. Specifically, all the Chinese evaluation tools incorporated in this study have demonstrated excellent reliability and validity, including content validity, construct validity, and internal consistency. However, aspects such as cross-cultural validity and measurement error have been scarcely mentioned, and the criterion validity has not been tested either. This situation might be attributed to the current absence of a unified theoretical framework for the self-management of hypertension.

Existing research has established self-management systems for elderly patients with chronic diseases, and the outcomes have been quite favorable [54]. Moreover, certain studies [55] have already elucidated the factors that influence the self-management concepts of patients with chronic diseases. Given the trend of younger onset of hypertension in China, it is feasible to conduct research on self-management systems for young patients with hypertension based on the current research achievements.

Currently, the research on self-management evaluation for patients with hypertension in China is still in the developmental stage, and the quality of self-management among Chinese patients with hypertension remains lower than that in developed countries. Therefore, it is imperative to continuously develop, refine, and optimize the evaluation tools by integrating national policies, cultural disparities, and the actual situation of healthcare. This will provide a solid foundation for Chinese clinical staff to implement interventions and treatment measures.

### Numerous assessment tools with varying characteristics

When their Cronbach’s α coefficients are ≥ 0.80, these instruments are considered to possess satisfactory internal consistency reliability; ICC ≥ 0.75 indicates good test-retest reliability. KMO and CVI serve as measures of construct validity and content validity, respectively, to assess the accuracy and logical coherence of evaluation tools. Reliability testing was conducted on all 20 instruments, among which the total scale and dimension item scores of four tools [32, 40–42] exceeded 0.7. Among the evaluation tools included in this study, the majority of those from China underwent reliability and validity testing, while only a few from other countries were subjected to reliability testing, which evidently restricts the promotion and application of these tools. The usability evaluation of assessment tools can refer to the methods mentioned in [56]. Currently, there is a lack of consensus and guidelines regarding the theoretical frameworks for self-management tools targeting hypertensive patients. In contrast, the Hypertension Self-Management Scale, the Hypertension Self-Management Behavior Assessment Scale, and the Treatment Adherence Questionnaire for Hypertensive Patients (TAQPH) align more closely with the objectives of hypertension self-management. The target populations for these evaluation tools vary; Wu’s [36] tool is designed for elderly patients, Gong’s [39] tool for hypertension risk screening, and Zheng’s [40] and Zhao’s [41] tools for hypertensive patients, all of which are multidimensional. The tool developed by Brocalaqui et al. [37] demonstrates good reliability results, features concise scale items and dimension numbers, and has a fixed evaluation cycle, yet it has not yet been introduced into China for localization adaptation.

### Limitations

Despite providing suitable tools for the assessment of hypertension self-management behaviors, this scoping review had several limitations. First, the study did not aim to investigate the established psychometric properties, which are crucial for ensuring the effectiveness and reliability of these tools in clinical practice. Future research should not only measure the reliability and validity of the tools but also focus on their psychometric characteristics. Second, by excluding gray literature, such as conference papers or dissertations, there is a potential to overlooking recently developed assessment tools. In addition, this review only considered the characteristics of the target populations for these tools and did not identify the assessment context, assessment tools, and evaluation of the quality of the literature for all tools. Despite these limitations, this review lays the groundwork for personalized self-management assessment of patients with hypertension in the future. The identified tools provide health care professionals with a starting point for selecting appropriate tools based on their specific needs and objectives. Further research should explore self-management assessment tools developed for different populations and hypertension conditions, making them applicable to patient groups in various settings. By understanding how to combine different assessment tools for maximum effectiveness, clinicians can develop more comprehensive, multidimensional, and personalized assessment tools for hypertension self-management. The ultimate goal is to provide personalized interventions or treatment plans for patients with hypertension.

## Conclusion

This study conducted a scoping review of instruments for assessing self-management in hypertensive patients, both domestically and internationally. The objective was to understand the current application status of these tools and provide a reference for future self-management assessment instruments for this population. Despite the relatively late start of research on hypertension self-management in China, several self-developed assessment tools have demonstrated good reliability and validity, making them suitable for various groups including the elderly, middle-aged, and hospitalized patients. However, pregnant women with hypertension have not received adequate attention, and assessment tools for this population have not undergone population trials abroad. Therefore, researchers are encouraged to avoid pitfalls in the development or localization of hypertension self-management assessment tools by following the COSMIN guidelines for rigorous tool validation. This approach will enhance the quality and applicability of the scales. In clinical settings, multidimensional tools that reflect the complexity of hypertension should be prioritized to comprehensively assess patients’ self-management abilities and inform intervention strategies.

## Electronic supplementary material

Below is the link to the electronic supplementary material.

Supplementary Material 1

Supplementary Material 2

Supplementary Material 3

Supplementary Material 4

Supplementary Material 5

## Data Availability

No datasets were generated or analysed during the current study.

## Declarations

### Provenance and peer review

Not commissioned; externally peer reviewed.
