# Supplementary material for: Self-management assessment tools for people with hypertension: a scoping review
Source: BMC Nephrol. 2025 Apr 30;26:219. doi: 10.1186/s12882-025-04134-y (PMC12044838; doi:10.1186/s12882-025-04134-y)
Supplement: Supplementary file 1 — Supplementary Material 1 [file 12882_2025_4134_MOESM1_ESM.docx]

# Appendix C – Search Strategy

The complete MEDLINE search strategy is shown below (Table 1). Database searches were conducted using a combination of subject terms plus free words related to hypertension, self-management, and assessment tools, and therefore searches were conducted in the title and abstract fields ([tiab]). Hypertension, were searched as per the above requirements and the results were in line 3 . Self-management and assessment tools were each searched in the same way, with all final search results centered on line 10. Standard MEDLINE methods were then used to remove animal studies, remove reviews and editorials (which were unlikely to report detailed results), and thus concentrate the results further down the list.

**Table A.1: MEDLINE (PubMed) strategy**

| **Search number** | **Query** | **Results** |
| --- | --- | --- |
| 10 | #3 AND #6 AND #9 | 2,278 |
| 9 | #7 OR #8 | 4,839,490 |
| 8 | (assessmen tool [Title/Abstract])OR(poll [Title/Ab- stract])OR (screening [Title/Abstract])OR (framework [Title/Abstract])OR (questionnaire [Title/Abstract])OR (scale [Title/Abstract]))OR (assessment [Title/Abstract])OR(Translati- on[Title/Abstract])OR(measuringinstrument[Title/Abstract])OR(interpret[Title/Abstract]) | 4,160,204 |
| 7 | Surveys and Questionnaires[Mesh] | 1,258,546 |
| 6 | #4 OR #5 | 192,006 |
| 5 | (Self-Administration[Title/Abstract])OR (Self-Management [Title/Abstract]))OR (Self-Care[Title/Abstract]))OR (self-Efficacy[Title/Abstract]))OR (self-Control[Title/Abstract]) | 113,454 |
| 4 | Self-Management[Mesh] | 120,710 |
| 3 | #1 OR #2 | 632,228 |
| 2 | Essential Hypertension[Title/Abstract]) OR (Isolated Systolic Hypertension[Title/Abstract]) OR (Hypertension,Malignant[Title/Abstract]) OR (Hypertension,Pregnancy-Induced[Title/Abstract]) OR (Hypertension,Pulmonary[Title/Abstract])OR (Hypertension,Renal[Title/Abstract])OR (Hypertension,Renovascular[Title/Abstract])OR (Hypertensive Crisis[Title/Abstract])OR (Hypertensive Retinopathy[Title/Abstract])OR (Retinal Arterial Macroaneurysm[Title/Abstract])OR (hypertensive[Title/Abstract])OR (high blood pressure[Title/Abstract])OR (chronic hypertension[Title/Abstract])OR (Chronic high blood pressure[Title/Abstract])OR (hypertensive disorder[Title/Abstract])OR (high blood pressure disease[Title/Abstract]) | 524497 |
| 1 | hypertension[Mesh] | 626107 |

# Database searches

The MEDLINE strategy was translated to run in each database as shown below.

**CNKI**

August 16th, 2024

**
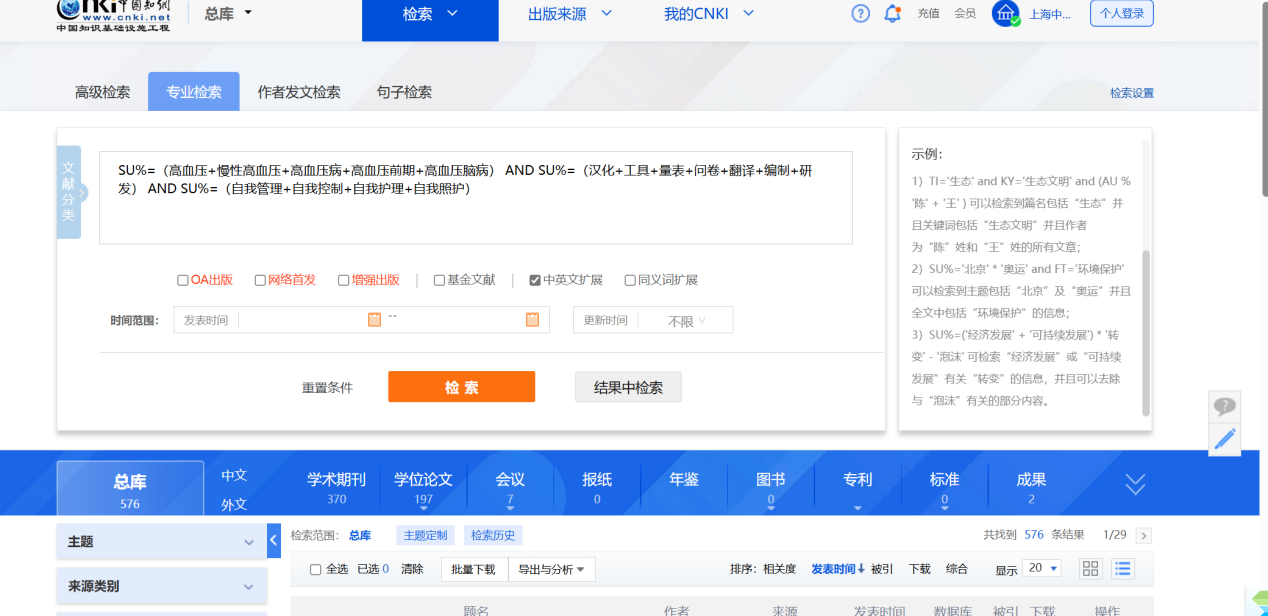
**

576 records were retrieved.

**VIP**

August 16th, 2024

**
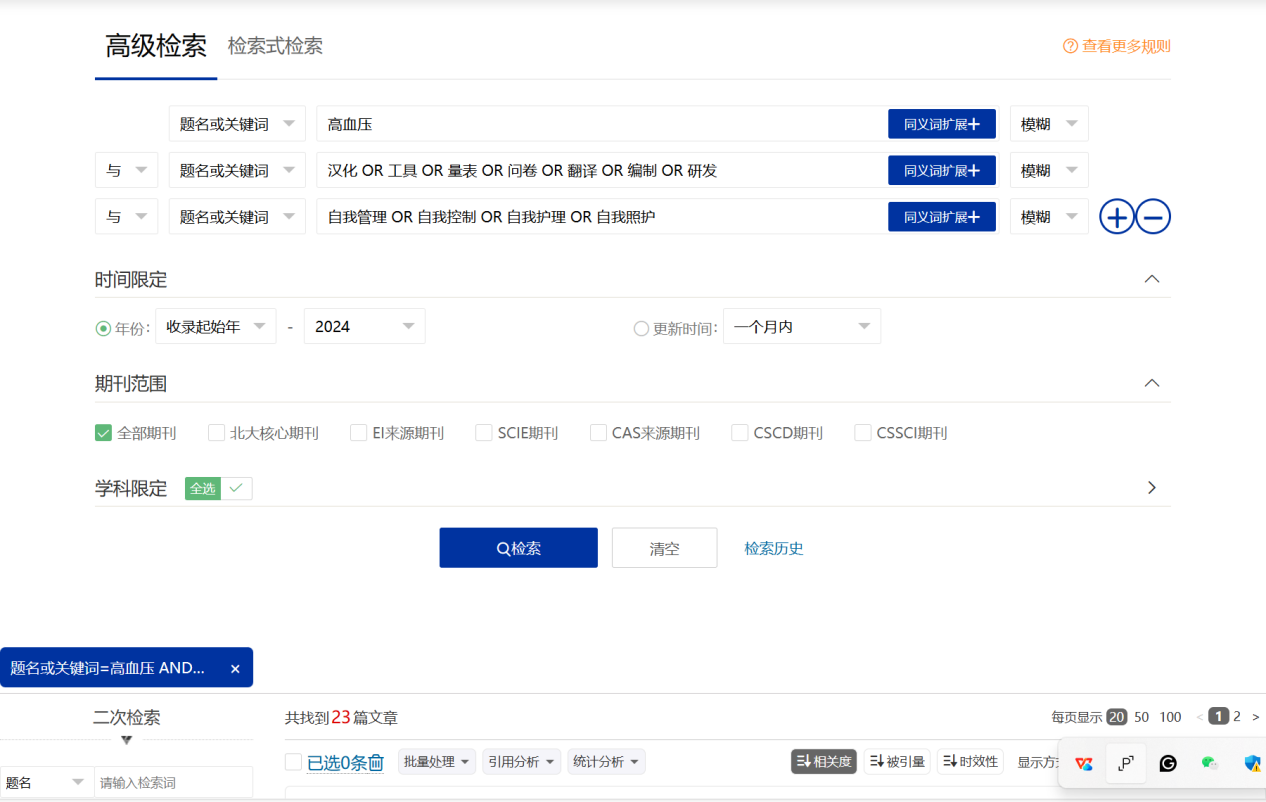
**

23records were retrieved.

**WANFANG**

August 16th, 2024

Excluding conference papers and dissertations, selecting literature for which full text is available:


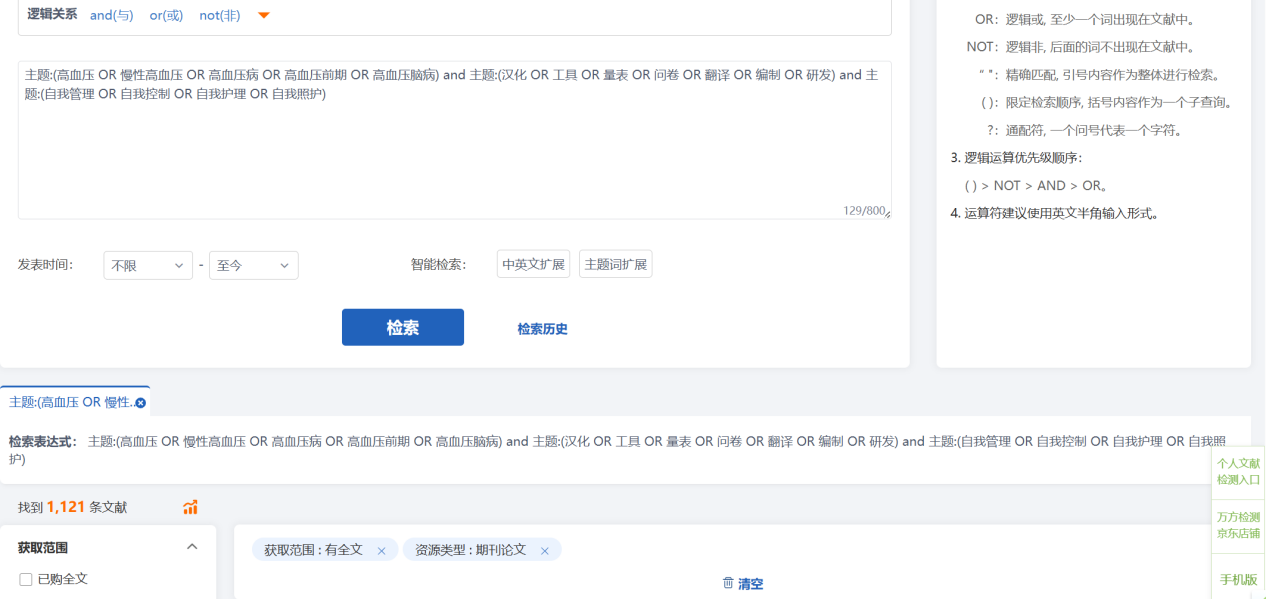


1121records were retrieved.

**CMB**

August 16th, 2024

The search strategy was:

Excluding conference papers and dissertations, selecting literature for which full text is available:


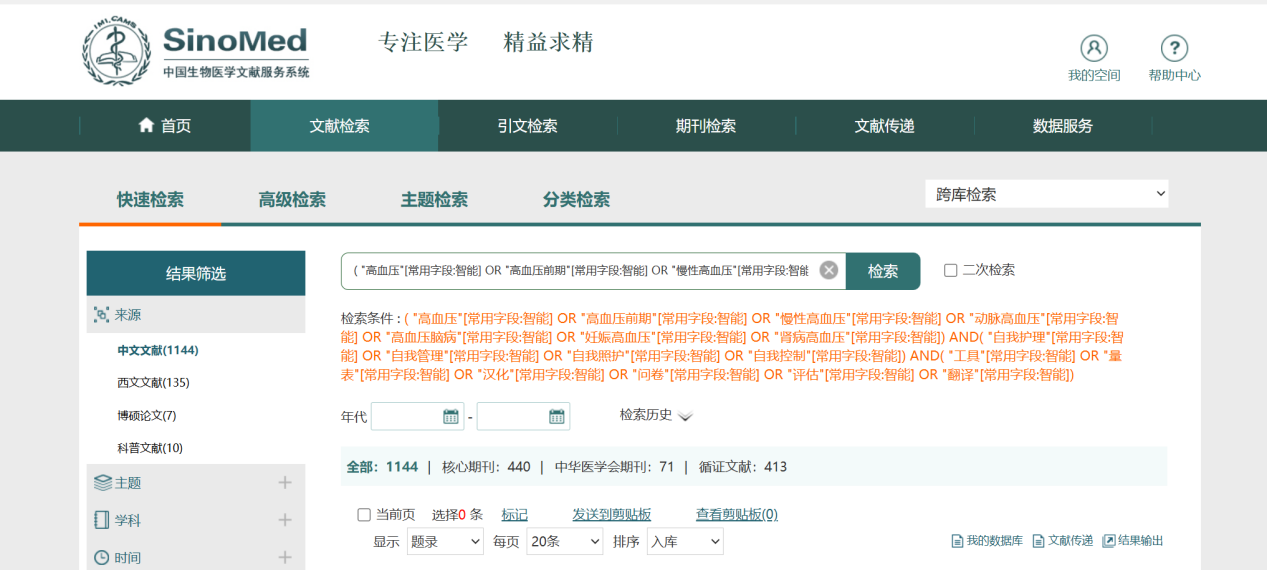


1279records were retrieved.

**PubMed**

August 17th, 2024

The search strategy was:

(((((Essential Hypertension[Title/Abstract]) OR (Isolated Systolic Hypertension[Title/Abstract] OR Hypertension,Malignant[Title/Abstract] OR Hypertension,Pregnancy-Induced[Title/Abstract] OR Hypertension,Pulmonary[Title/Abstract] OR Hypertension,Renal[Title/Abstract] OR Hypertension,Renovascular[Title/Abstract] OR Hypertensive Crisis[Title/Abstract] OR Hypertensive Retinopathy[Title/Abstract] OR Retinal Arterial Macroaneurysm[Title/Abstract] OR hypertensive[Title/Abstract] OR high blood pressure[Title/Abstract] OR chronic hypertension[Title/Abstract] OR Chronic high blood pressure[Title/Abstract] OR hypertensive disorder[Title/Abstract] OR high blood pressure disease[Title/Abstract] OR hypertension[Title/Abstract])) OR (hypertension)) AND ((Self-Management) OR (Self-Administration[Title/Abstract] OR Self-Management[Title/Abstract] OR Self-Care[Title/Abstract] OR self-Efficacy[Title/Abstract] OR self-Control[Title/Abstract]))) AND ((assessmen tool[Title/Abstract] OR poll[Title/Abstract] OR screening[Title/Abstract] OR framework[Title/Abstract] OR questionnaire[Title/Abstract] OR scale[Title/Abstract] OR assessment[Title/Abstract] OR Translation[Title/Abstract] OR measuring[Title/Abstract] OR instrument[Title/Abstract] OR interpret[Title/Abstract]) OR (Surveys and Questionnaires)))

2,278  records were retrieved.

**Web of Science**

August 17th, 2024

|  |  |  |
| --- | --- | --- |
| #1 | surveys* OR questionnaires* OR assessment tool* OR screening OR scale OR Translation OR measuring OR instrument |  |
| #2 | Hypertension* or High Blood Pressure or Hypertensive or High Blood Pressures or Blood Pressure, High Essential Hypertension OR Isolated Systolic Hypertension OR Hypertension,Malignant OR Hypertension,Pregnancy-Induced OR Hypertension,Pulmonary OR Hypertension,Renal OR Hypertension,Renovascular OR Hypertensive Crisis OR Hypertensive Retinopathy OR Retinal Arterial Macroaneurysm OR hypertensive* OR high blood pressure* OR chronic hypertension OR Chronic high blood pressure OR hypertensive disorder OR high blood pressure disease OR hypertension* OR hypertension |  |
| #3 | Self-Administration OR Self-Management OR Self-Care OR self-Efficacy OR self-Control |  |
| #4 | #1 AND #2 AND #3 | 3746 |


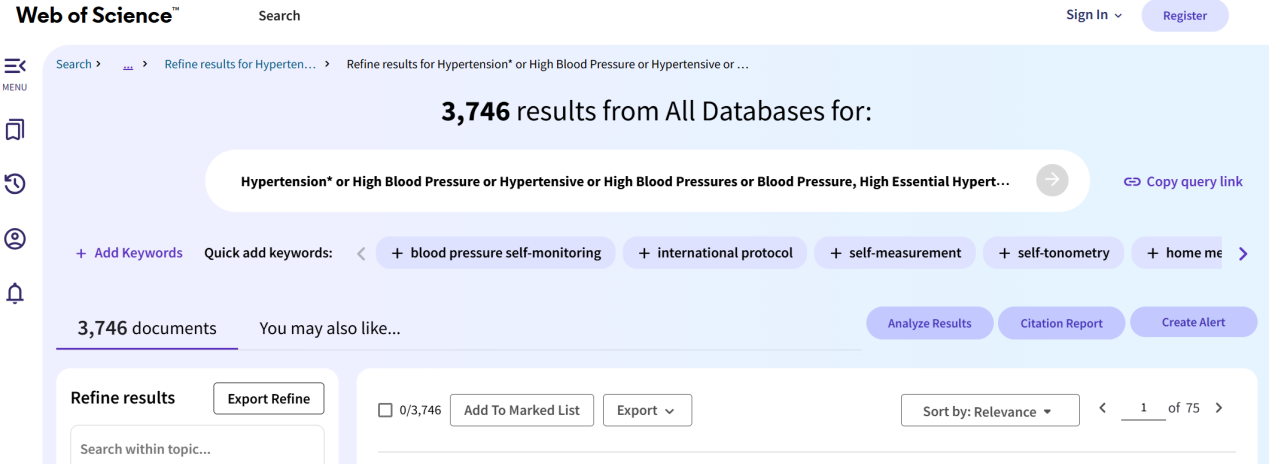


3746 records were retrieved.

**CINAHL (via EBSCOHost)**

August 17th, 2024

**
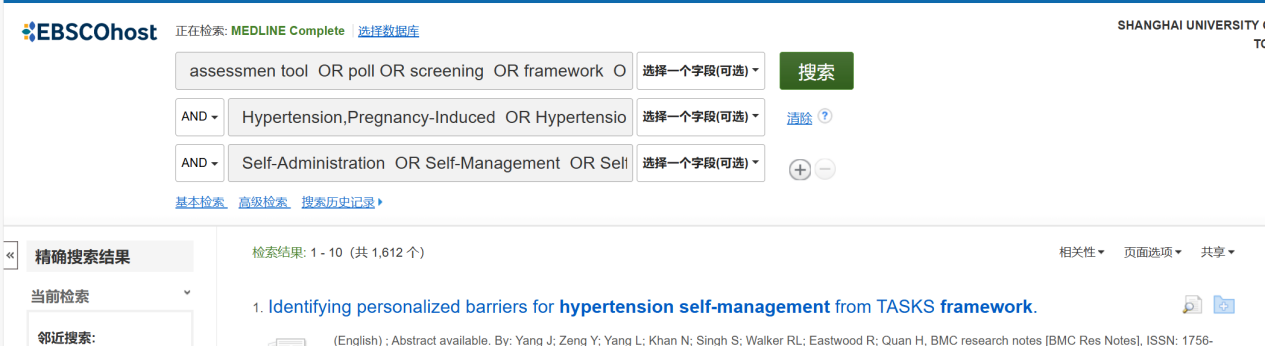
**

**1612** records were retrieved.

**Scopus**

August 17th, 2024


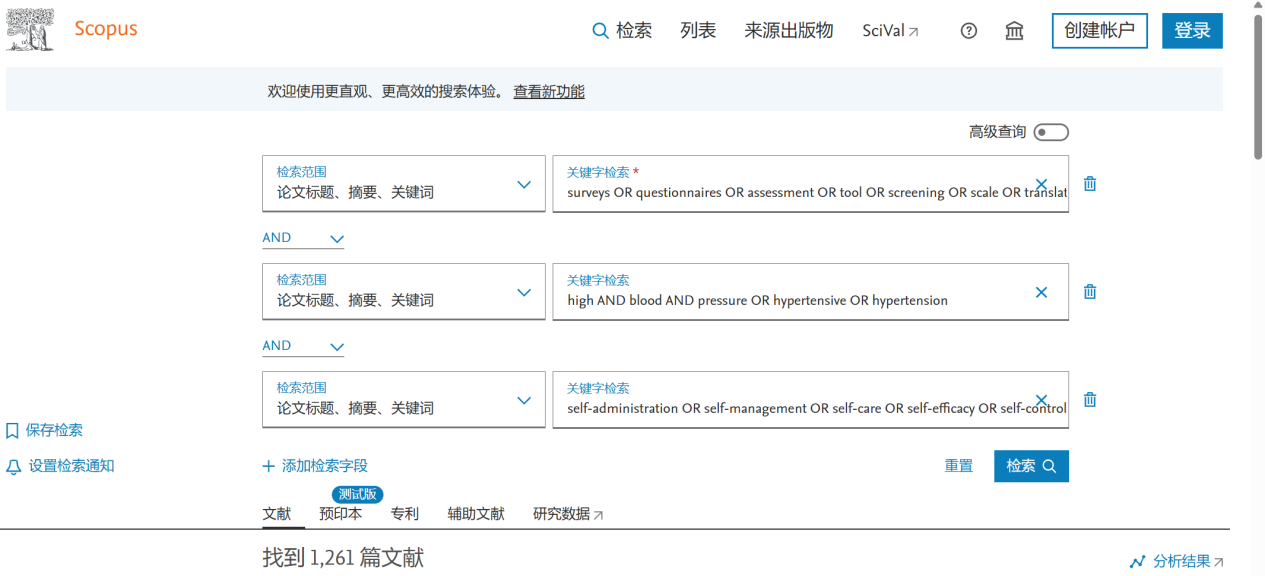


**1261** records were retrieved.

**Cochrane Library**

August 17th, 2024

**
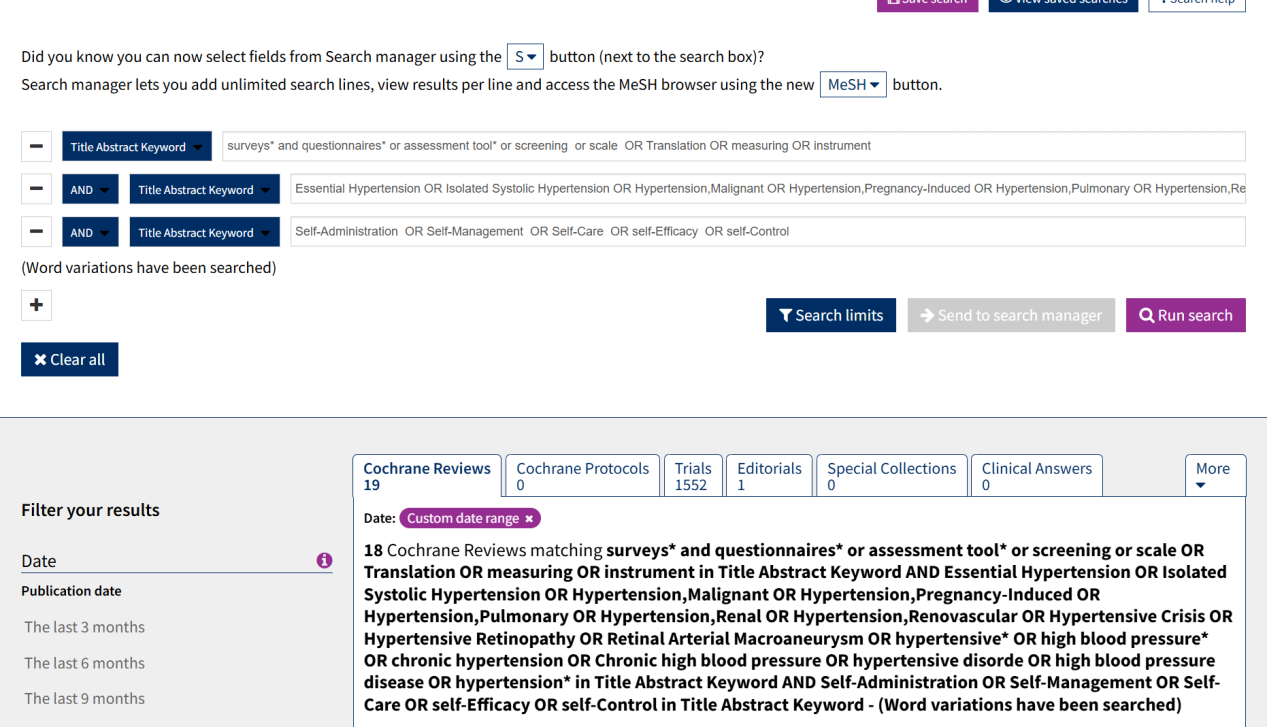
**

**1571** records were retrieved.

**Epistemonikos**

August 17th, 2024

Without access to this database, we can't retrieve it.

**Many foreign databases don't have access in China and we can't search them.**

**Table A.2: Number of records retrieved from databases and other resources**

| **Database/resource** | **Date searched** | **Number of records downloaded** | **Number of records remaining after deduplication** |
| --- | --- | --- | --- |
| **CNKI** | 2024/08/16 | **576** | **541** |
| **VIP** | 2024/08/16 | 23 | 5 |
| **WANFANG** | 2024/08/16 | 1121 | 527 |
| **CMB** | 2024/08/16 | 1279 | 56 |
| **PubMed** | 2024/08/17 | 2,278 | 1849 |
| **Web of Science** | 28/7/22 | 3746 | 1931 |
| **Scopus** | 28/7/22 | 1261 | 21 |
| **CINAHL (via EBSCOHost)** | 24/7/22 | 1612 | 0 |
| **Cochrane Library** | 24/7/22 | 1571 | 16 |
