# Supplementary material for: Self-management assessment tools for people with hypertension: a scoping review
Source: BMC Nephrol. 2025 Apr 30;26:219. doi: 10.1186/s12882-025-04134-y (PMC12044838; doi:10.1186/s12882-025-04134-y)
Supplement: Supplementary file 2 — Supplementary Material 2 [file 12882_2025_4134_MOESM2_ESM.docx]

Excluded studies with reasons for exclusion

The reasons for the exclusion of assessment tools are standardized below:

Reason 1 = No methodology or theoretical basis for construction

Reason 2 = Does not fit or apply to a wide range of people

Reason 3 = Full text not available.

Reason 4 = Translated version.

Shown as “Report not retrieved” in the PRISMA flowchart and not used as a reason for exclusion.

| References | Reason |
| --- | --- |
| Van Truong P , Lin M Y , Chiu H Y ,et al.Psychometric Properties and Factorial Structure of Vietnamese Version of the Hypertension Self-Care Profile Behavior Scale[J].Journal of Cardiovascular Nursing, 2020, publish ahead of print.DOI:10.1097/JCN.0000000000000770. | 4 |
| Alsaqer K , Bebis H .Cross-Cultural Adaptation, Validity, and Reliability of the Arabic Version of the Self-care of Hypertension Inventory Scale Among Older Adults[J].The Journal of cardiovascular nursing, 2020, Publish Ahead of Print.DOI:10.1097/JCN.0000000000000738. | 4 |
| Helena E T D S , Nemes M I B ,José Eluf-Neto.[Development and validation of a multidimensional questionnaire assessing non-adherence to medicines].[J].Faculdade de Saúde Pública da Universidade de São Paulo, 2008(4).DOI:10.1590/S0034-89102008000400025. | 2 |
| Steeg N V D , Sielk M , Pentzek M ,et al.Drug-adherence questionnaires not valid for patients taking blood-pressure-lowering drugs in a primary health care setting.[J].Journal of Evaluation in Clinical Practice, 2010, 15(3):468-472.DOI:10.1111/j.1365-2753.2008.01038.x. | 1 |
| McHorney C A. The Adherence Estimator: a brief, proximal screener for patient propensity to adhere to prescription medications for chronic disease[J]. Current medical research and opinion, 2009, 25(1): 215-238.DOI: 10.1185/03007990802619425. | 2 |
| Johnson MJ, Rogers S. Development of the purposeful action medication-taking questionnaire. West J Nurs Res. 2006 Apr;28(3):335-51. DOI: 10.1177/0193945905284726. | 2 |
| Witoniowska-Lonc N ,Jacek Polański,Beata Jankowska-Polańska.Psychometric Properties of the Polish Version of the Self-care of Hypertension Inventory.[J].Ovid Technologies (Wolters Kluwer Health), 2021(5).DOI:10.1097/jcn.0000000000000776. | 4 |
| Lambert E V , Steyn K , Stender S ,et al.Cross-cultural validation of the hill-bone compliance to high blood pressure therapy scale in a South African, primary healthcare setting.[J].Ethnicity & disease, 2006, 16(1):286-291.DOI:10.1016/S0895-7061(02)02817-0. | 4 |
| Al-Noumani H S, Al Omari O. Psychometric Properties of the Arabic Version of Medication Adherence Self-Efficacy Scale-Revised in Hypertension[J]. Journal of Nursing Measurement, 2022, 30(1).DOI: 10.1891/JNM-D-20-00055. | 4 |
| Ogedegbe G , Mancuso C A , Allegrante J P ,et al.Development and evaluation of a medication adherence self-efficacy scale in hypertensive African-American patients[J].Journal of Clinical Epidemiology, 2003, 56(6):520-529.DOI:10.1016/S0895-4356(03)00053-2. | 1 |
| Ngoh S H A , Lim H W L , Koh Y L E ,et al.Test–retest reliability of the Mandarin versions of the Hypertension Self-Care Profile instrument[J].Medicine, 2017, 96(45):e8568.DOI:10.1097/MD.0000000000008568. | 4 |
| Svarstad B L, Chewning B A, Sleath B L, et al. The Brief Medication Questionnaire: a tool for screening patient adherence and barriers to adherence[J]. Patient education and counseling, 1999, 37(2): 113-124.DOI: 10.1016/s0738-3991(98)00107-4. | 2 |
| Jun,Younghee,Song,et al.Psychometric Properties of the Hypertension Self-Care Behavior Scale for Elders with Hypertension in Korea[J].Journal of the Korean Academy of Fundamentals of Nursing, 2017, 24(1):1-8. | 4 |
| Kripalani S, Risser J, Gatti M E, et al. Development and evaluation of the Adherence to Refills and Medications Scale (ARMS) among low-literacy patients with chronic disease[J]. Value in Health, 2009, 12(1): 118-123.DOI: 10.1111/j.1524-4733.2008.00400.x. | 2 |
| Metin Z G, Eren M G, Ozsurekci C, et al. Turkish validity and reliability of the self-care of hypertension inventory (sc-hi) among older adults[J]. Journal of Community Health Nursing, 2023, 40(1): 64-77.DOI: 10.1080/07370016.2022.2078163. | 4 |
| Phonphet C, Suwanno J, Thiamwong L, et al. Translation and cross-cultural adaptation of the self-care of hypertension inventory for Thais with hypertension[J]. Journal of Cardiovascular Nursing, 2023, 38(2): 179-191. DOI: 10.1097/JCN.0000000000000895. Epub 2022 Jan 31. | 4 |
| Moharamzad Y, Saadat H, Shahraki B N, et al. Validation of the Persian version of the 8-item Morisky Medication Adherence Scale (MMAS-8) in Iranian hypertensive patients[J]. Global journal of health science, 2015, 7(4): 173.DOI: 10.5539/gjhs.v7n4p173. | 3 |
| Herrera Guerra E P, Robles González J R, Bautista Arellano L R. Validez y confiabilidad del Self-Care of Hypertension Inventory, versión español colombiano[J]. Avances en Enfermería, 2021, 39(2): 215-224. | 4 |
| Shabani M, Taheri-Kharameh Z, Sheikholeslamikabiri F, et al. Translation and evaluation of psychometric properties of the Persian version of the Hypertension Self-Care Activity Level Effects (H-SCALE)[J]. BMC Cardiovascular Disorders, 2023, 23(1): 422.DOI: 10.1186/s12872-023-03460-z. | 4 |
| Teng, Z., Sun, Z. & Zhou, C. (2008). Analysis of knowledge, behavior, and influencing factors related to hypertension patients. Journal of Nursing, 15(8), 3. DOI: CNKI:SUN:NFHL.0.2008-08-010. | 3 |
| Liu, J. & Zhao, Y. (2008). Evaluation of nursing intervention effects on patients with primary hypertension under ambulatory blood pressure monitoring. Nursing Research, 22(10), 2588-2589. DOI: 10.3969/j.issn.1009-6493.2008.28.025. | 3 |
