# Supplementary material for: Self-management assessment tools for people with hypertension: a scoping review
Source: BMC Nephrol. 2025 Apr 30;26:219. doi: 10.1186/s12882-025-04134-y (PMC12044838; doi:10.1186/s12882-025-04134-y)
Supplement: Supplementary file 3 — Supplementary Material 3 [file 12882_2025_4134_MOESM3_ESM.docx]

**Identification of studies via databases and registers**

**Identification**

Records identified through databases(n=10458):

CKNI=598、VIP=21、WANFANG=1602、CMB=1085、PubMed=2130、Web of Science=2014、Embase=226、Cochrane Library=832、Scupos=1950

Duplicates were removed(n=6521)

Articles were exclude（n=3,579)

Titles and abstracts were screened(n=3937)

Additional records identified through bibliographic search(n=12)

**Screening**

Full texts were read(n=358).

Articles were excluded（n=329):

- irrelevant topic (n=217)
- Not original research(n=76)
- Not in Chinese or English(n=6)
- Full text unavailable（n=19)
- Reviews or conference abstracts (n=11)

Studies included in review (n=41)

**Included**

Extracted assessment tools(n=20)

*Consider, if feasible to do so, reporting the number of records identified from each database or register searched (rather than the total number across all databases/registers).

**If automation tools were used, indicate how many records were excluded by a human and how many were excluded by automation tools.

13467 – 6521 = 6946. So, the ‘Titles and abstracts were screened’ should be 6946

I have re-counted the statistics
