# Supplementary material for: Self-management assessment tools for people with hypertension: a scoping review
Source: BMC Nephrol. 2025 Apr 30;26:219. doi: 10.1186/s12882-025-04134-y (PMC12044838; doi:10.1186/s12882-025-04134-y)
Supplement: Supplementary file 5 — Supplementary Material 5 [file 12882_2025_4134_MOESM5_ESM.pdf]

浦东新区卫生健康委员会
学科建设计划任务合同书

学科名称：急救护理学

类　　别：重要薄弱学科

建设周期：2023-1-1 至 2026-12-31

单位名称：上海市浦东新区人民医院　（盖章）

通讯地址：上海市浦东新区川环南路 490 号

邮政编码：201299　联系电话：021-20509048

学科负责人：江萍

联系人手机：13621762508　E-Mail：1343840628@qq.com

填写日期：2022-7-28

PWZbr2022-10

浦东新区卫生健康委员会
二〇二〇年

一、基本情况表

1、单位情况

单位名称	上海市浦东新区人民医院		
通讯地址	上海市浦东新区川环南路 490 号	邮编	201299
科研部门联系人	周雪	联系电话	021-20509048
电子邮件	ky@shpdph.com	传真	021-58981219
财务部门联系人	马煜	联系电话	021-20509085
账户名	上海市浦东新区人民医院	开户银行	上海银行川沙支行
银行账号	03004344088		

2、学科情况

学科名称	急救护理学		
床位数	ICU25 张/急诊病区 40 张/急诊暂观 3	人员数	88
年门诊人次	41 万	年出院人次	600
联系人	江萍	联系电话	13621762508
电子邮件	1343840628@qq.com	传真	021-58981219

3、学科负责人情况

姓名	江萍	性别	女	出生年月	1968-8-24
最高学历	本科	学位	学士学位	获得时间	1990-7-15
授予院校	上海第二医科大学				
研究生导师	☐博导　☑硕导　☐非导师				
工作部门	护理部		行政职务	护理部主任、社会服务部主任	
从事专业	护理		职　称	主任护师	

五、人员信息表

序号	姓名	年龄	学位	技术职称	所在单位	科室	专业	签字
1	江萍	54	学士学位	主任护师	上海市浦东新区人民医院	护理部	护理管理、护理心理、急救护理、慢病护理	江萍
2	曹英华	46	无	主管护师	上海市浦东新区人民医院	护理部	护理教育、慢病护理	曹英华
3	杜锦萍	51	无	主管护师	上海市浦东新区人民医院	急诊科	急救/重症护理、护理教育	杜锦萍
4	周艳	50	无	副主任护师	上海市浦东新区人民医院	护理部	急救/重症护理、外科护理、PICC	周艳
5	张伟	38	学士学位	主管护师	上海市浦东新区人民医院	急诊科	急救/重症护理	张伟
6	范群	35	无	主管护师	上海市浦东新区人民医院	急诊科	急救/重症护理	范群
7	沈晓玲	36	无	主管护师	上海市浦东新区人民医院	急诊科	急救/重症护理	沈晓玲
8	张金换	40	无	主管护师	上海市浦东新区人民医院	急诊科	急救/重症护理	张金换
9	王海燕	33	学士学位	护师	上海市浦东新区人民医院	急诊科	急救/重症护理	王海燕
10	张慧丽	41	无	护师	上海市浦东新区人民医院	急诊科	急救/重症护理	张慧丽
11	陶燕	32	无	护师	上海市浦东新区人民医院	急诊科	急救/重症护理	陶燕
12	洪叶	30	无	护师	上海市浦东新区人民医院	急诊科	急救/重症护理	洪叶
13	付迪	26	学士学位	护师	上海中医药大学研究生院、上海市浦东新区人民医院	护理部	慢病管理、急救护理	付迪
14	李梦较	24	学士学位	护师	上海中医药大学研究生院、上海市浦东新区人民医院	护理部	慢病管理、急救护理	李梦较
15	张丽娜	36	无	护师	上海市浦东新区人民医院	急诊科	急救/重症护理	张丽娜
16	徐欢	40	无	护师	上海市浦东新区人民医院	急诊科	急救/重症护理	徐欢
17	周静芬	42	无	副主任护师	上海市浦东新区人民医院	骨科	骨科护理、中医护理	周静芬

18	奚凤群	43	无	副主任护师	上海市浦东新区人民医院	心内科	心内科护理、PICC	
19	王燕	32	学士学位	主管护师	上海市浦东新区人民医院	教学办	护理教育、慢病护理	
20	凤春燕	43	无	主管护师	上海市浦东新区人民医院	普外科	外科护理、危重症护理	
21	宁玉萍	34	学士学位	护师	上海市浦东新区人民医院	护理部	慢病护理、GCP	
22	黄丽文	32	学士学位	护师	上海市浦东新区人民医院	护理部	急救/重症护理	
23	段文杰	35	博士学位	教授	华东理工大学	社会工作系	社会心理服务体系与健康心理服务体系建设研究	
24	史燕伟	31	硕士学位	副教授	上海师范大学	组织行为与人力资源管理	组织行为与人力资源管理	

总人数	正高	副高	中级	初级	其他	
	2	4	6	12	0	
	硕博导	博士	硕士	学士	其他	
24	3	1	1	8	14	

PWZbr2022-10

九、单位意见

1. 对学科建设、经费预算及能否保证计划实施所需人、物、财力等的承诺，单位法人签名盖单位公章<br><br>单位负责人签章<br>公　章<br>年　月　日
2. 参与单位审查意见与保证（同意参加协作，保证对参加合作人员时间、工作条件及经费的支持、督促其按计划完成所承担的任务等）<br><br>参与单位　　　　　　　　参与单位<br>单位负责人签章　　　　　单位负责人签章<br>公　章　　　　　　　　　公　章<br>年　月　日　　　　　　　年　月　日

PWZbr2022-10

十、浦东新区卫生健康委员会意见

上海市浦东新区卫生健康委员会

单位负责人签章

公 章

年 月 日

PWZbr2022-10
